# Employment of Muriate of Ammonia in Scirrhus of the Stomach

**Published:** 1843-06

**Authors:** 


*Employment of Muriate of Ammonia in Scirrhus of the Stomach.*—The above salt having been administered by German practitioners with decided benefit in cases of induration and degenerescence of the bladder, prostate gland, &c., Trusen was induced to employ it in the following instances:

Case 1.—A man, thirty years of age, accustomed to a sedentary life, and the too great use of alcoholic liquors and strongly spiced aliments, had evidently contracted a scirrhous disease of the pyloric extremity of the stomach. For some months, vomiting, with violent heartburn, &c., had always come on from three to four hours after taking food. Ordered, ammon. hydrochl. fifteen grains, every two hours, combined with extract of liquorice. In a short time digestion was performed better, and the appetite restored. Care and abstemiousness in diet were enjoined, and at the end of a six months' persistence in the use of the medicine, the vomitings ceased entirely. A clogged state of the stomach, from superabundance of mucus and habitual constipation, were removed by a six weeks visit to the muriated-chalybeate springs of Cudova, and the man subsequently regained perfect health.

Case 2.—A man of age, and sedentary habits, similar to the foregoing, had been for some time affected with arthritis, &c., on the removal of which he began to be subject to an invariable rejection of the whole of his food a few hours after it had been taken, accompanied by atrophy and emaciation. He entered, however, upon the use of muriate of ammonia in drachm doses (!) which he continued for seven months, when his vomitings had wholly and permanently disappeared.

The system of the patient soon adapted itself without inconvenience to the above large doses of the remedy, which were taken in infusion of ginger.—*Lond. Lancet*, from *Hufeland's Journal* and *Gaz. des Hôpitaux.*

